# A rare complication of blood culture-negative infective endocarditis on tricuspid valve: case report

**DOI:** 10.1093/ehjcr/ytae570

**Published:** 2024-10-22

**Authors:** Giovanni Bellina, Salvatore Scandura, Salvatore Lentini, Davide Capodanno, Corrado Tamburino

**Affiliations:** Azienda Ospedaliera Universitaria Policlinico ‘G. Rodolico-San Marco’, Via S. Sofia 78, 95100 Catania, Italy; Azienda Ospedaliera Universitaria Policlinico ‘G. Rodolico-San Marco’, Via S. Sofia 78, 95100 Catania, Italy; Azienda Ospedaliera Universitaria Policlinico ‘G. Rodolico-San Marco’, Via S. Sofia 78, 95100 Catania, Italy; Azienda Ospedaliera Universitaria Policlinico ‘G. Rodolico-San Marco’, Via S. Sofia 78, 95100 Catania, Italy; Azienda Ospedaliera Universitaria Policlinico ‘G. Rodolico-San Marco’, Via S. Sofia 78, 95100 Catania, Italy

**Keywords:** Blood culture-negative infective endocarditis, tricuspid valve, cardiac surgery, transoesophageal echocardiogram, case report

## Abstract

**Background:**

Endocarditis is an infectious disease, with an incidence of ∼15 cases per 100 000 people, affecting the tricuspid valve in 10% of cases. Infective endocarditis with negative blood cultures (BCNIE) accounts for more than 20% of cases of infective endocarditis. Perivalvular extension of the infection represents the most detrimental complications of BCNIE.

**Case summary:**

A 25-year-old South Asian male was admitted due to fever for 15 days and new onset chest pain. The blood tests showed an increase in inflammatory indices. A chest X-ray showed enlargement of the cardiac shadow. On cardiac examination, a holosystolic murmur at the left sternal edge border was heard. The transthoracic echocardiogram showed a filamentous formation on the tricuspid valve and communication between the aorta and right atrium with left–right shunt. A transoesophageal echocardiogram (TEE) was performed to confirm the diagnosis of IE. Three sets of blood cultures were performed, with negative results, empirical therapy was managed and a decision for TEE-guided cardiac surgery was made.

**Discussion:**

Fistula is a rare complication of IE representing the most insidious consequence of uncontrolled infection like BCNIE, a condition that has restricted the therapeutic possibilities to empirical therapy only and to early surgery. The TEE was instrumental in diagnosing right-sided infective endocarditis and allowing us to focus on the perivalvular spread of the infection in our case.

**Learning points**

- Complicated blood culture-negative infective endocarditis of the tricuspid valve is a rare condition that restricts the therapeutic possibilities.
- The transoesophageal echocardiogram played a key role in diagnosing right-sided infective endocarditis and helping us focus on the spread of the infection.

## Introduction

Endocarditis is an infectious disease with an incidence of ∼15 cases per 100 000 people, mainly affecting the left sections of the heart (mitral and aortic valves in 90%) and in 10% of cases also the right side.^1,2^ Infective endocarditis with negative blood cultures (BCNIE) accounts for more than 20% of cases of infective endocarditis representing a diagnostic and therapeutic challenge.^3^ The main agents responsible for this type of endocarditis are fungi and obligate intracellular bacteria.^3^ Among the urgent and most detrimental complications of BCNIE, perivalvular extension of the infection plays a major role.^4^ The diagnosis is based on the modified Dukes criteria (2015) comprising major and minor criteria among which blood cultures, serological tests, echocardiogram, and cardiac CT scan play a major role. The therapeutic approach consists of drug therapy and, if indicated, cardiac surgery.^4^

## Summary figure

**Table:** 

Presentation	Fever for 15 days and new onset chest pain, followed by blood test, chest X-ray, electrocardiogram, transthoracic echocardiogram.
Day 1	Transoesophageal echocardiogram and blood cultures to confirm infective endocarditis. Empirical therapy with beta-lactam and glycopeptide antibiotics.
Day 6	Negative blood cultures, continued empirical therapy.
Day 10	Cardiac surgery.
Day 15	Fever resolution.
Day 25	Transoesophageal echocardiogram showed mild tricuspid insufficiency and mild left–right shunt.
Day 45	Clinical recovery, discharge to cardiac rehabilitation.
Day ∼90	Transoesophageal echocardiogram showed a result comparable to the post-operative examination.

## Case presentation

**Figure 1:**
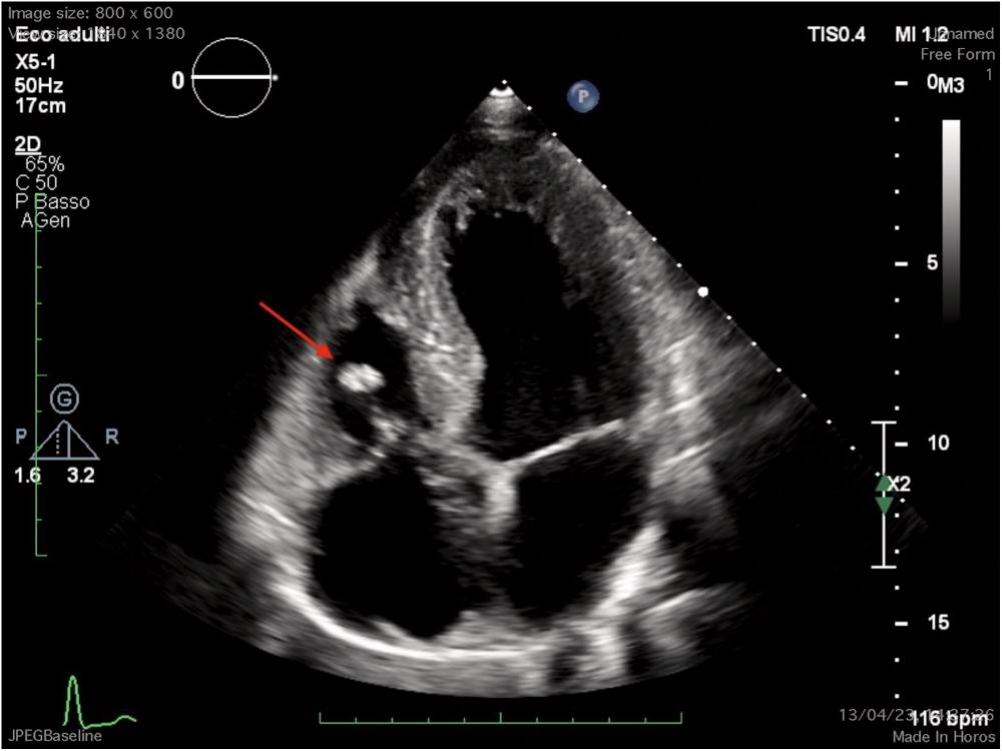
Transthoracic echocardiogram (apical four-chamber), vegetation on the tricuspid valve (arrow).

**Figure 2:**
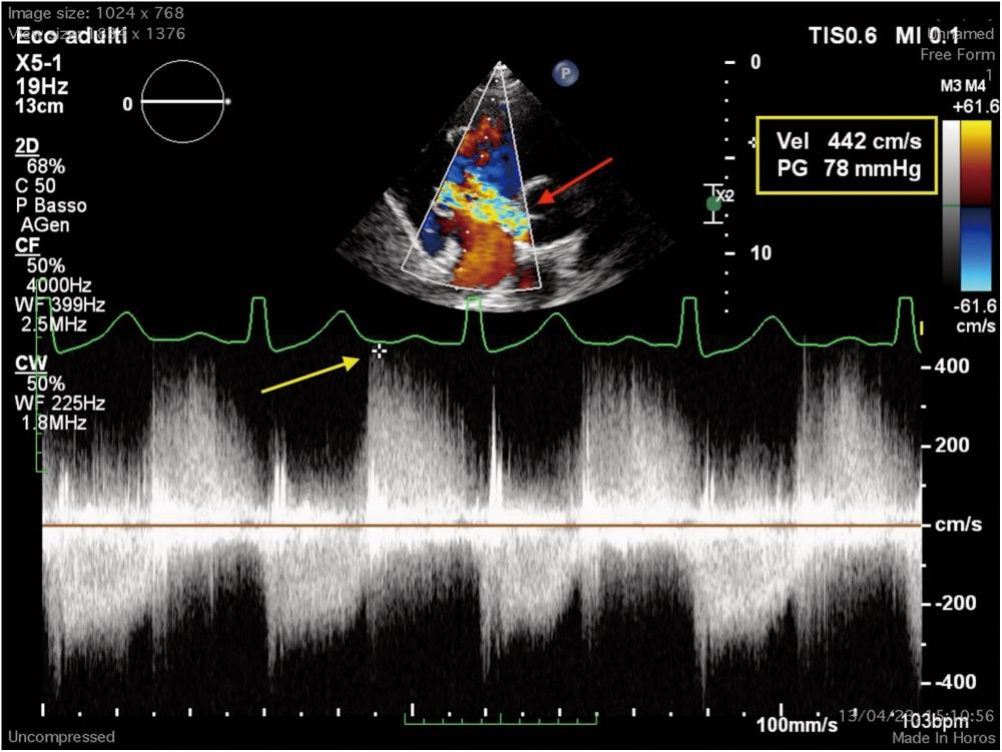
TTE (short axis), colour to study fistula and left–right shunt (red arrow), Doppler to study shunt’s maximum velocity and gradient (yellow box and yellow arrow).

A 25-year-old South Asian male was admitted due to fever for 15 days and new onset chest pain. He denied drug use, and his past medical history was non-contributory. He underwent blood tests which showed an increase in inflammatory indices [WBC 15.7 × 10^3^ ηg/L (n.r. 4.5–11 × 10^3^ ηg/L), C-reactive protein 25 mg/dL (n.r. 0.3–1 mg/dL), ESR 118 mm/h (0–15 mm/h)] but normal liver and kidney function. An electrocardiogram showed non-specific alterations in the ventricular repolarization phase, and a chest X-ray showed left basal pleural thickening, pleural effusion and enlargement of the cardiac shadow. On cardiac examination, a holosystolic murmur at the left sternal edge border was heard. Respiratory and abdominal examination was normal. Blood pressure was 120/80 mmHg, heart rate 75 beats per minute, and respiratory rate 12 breaths per minute. The transthoracic echocardiogram showed a filamentous formation on the posterior leaflet of the tricuspid valve (*Figure 1*), a broken chordae tendineae with flail of the anterior leaflet and communication between the aorta and right atrium with severe left–right shunt (maximum gradient 80 mmHg) (*Figure 2*); the remaining parameters were normal (left ventricular ejection fraction 55%). Therefore, once the diagnostic question of infective endocarditis had been posed, the procedure continued with three sets of blood cultures to search for the main aerobic and anaerobic microorganisms responsible for infective endocarditis (*Staphylococcus areus*, *Streptococcus viridans*, *Streptococcus bovis*, Enterococci, HACEK group), with serological tests to search for less common microorganisms (*Coxiella burnetii*, *Bartonella* spp., *Aspergillus* spp., *Mycoplasma pneumoniae*, and *Legionella pneumophila*), with specific polymerase chain reaction assays for *Tropheryma whipplei*, *Bartonella* spp., and fungi (*Candida* spp., *Aspergillus* spp.) from blood, and finally, with the assay of anti-nuclear antibodies, anti-cardiolipin antibodies (IgG), and anti-β2-glycoprotein 1 (IgG, IgM) with the aim of ruling out an anti-phospholipid antibody syndrome. Pending this assessment, empirical therapy with piperacillin/tazobactam 4.5 g 3/day + vancomycin 500 mg 2/day was administered.

**Figure 3:**
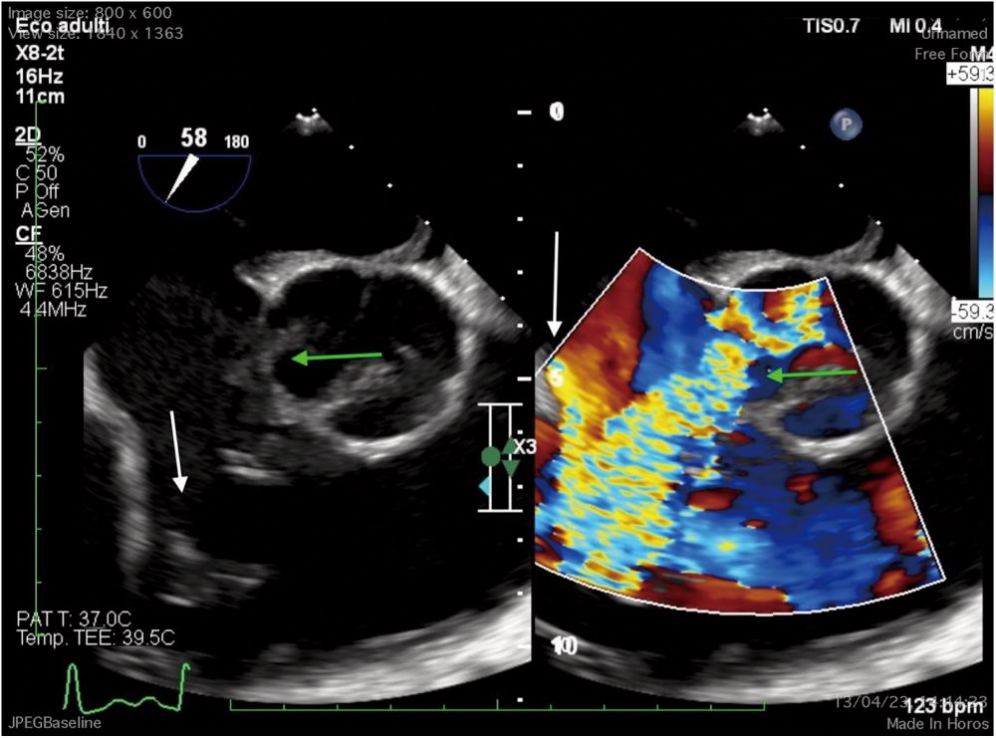
TEE (short axis), tricuspid regurgitation (white arrow) and aorta-right atrium’s shunt (green arrow) studied with colour Doppler.

A transoesophageal echocardiogram (TEE) was performed, which showed a large tricuspid anterior leaflet flail, a mobile formation on the atrial side of the septal leaflet and a small formation on the posterior leaflet, which led to severe insufficiency with multiple jets; in addition, there was evidence of a tricuspid aortic valve with fissuring (∼9 mm) at the level of the left coronary cusp determinant of a large shunt between the aorta and right atrium (*Figure 3*). Finally, a search for foci of systemic embolization was performed by abdomen–chest–brain CT scan, with negative results.

**Figure 4:**
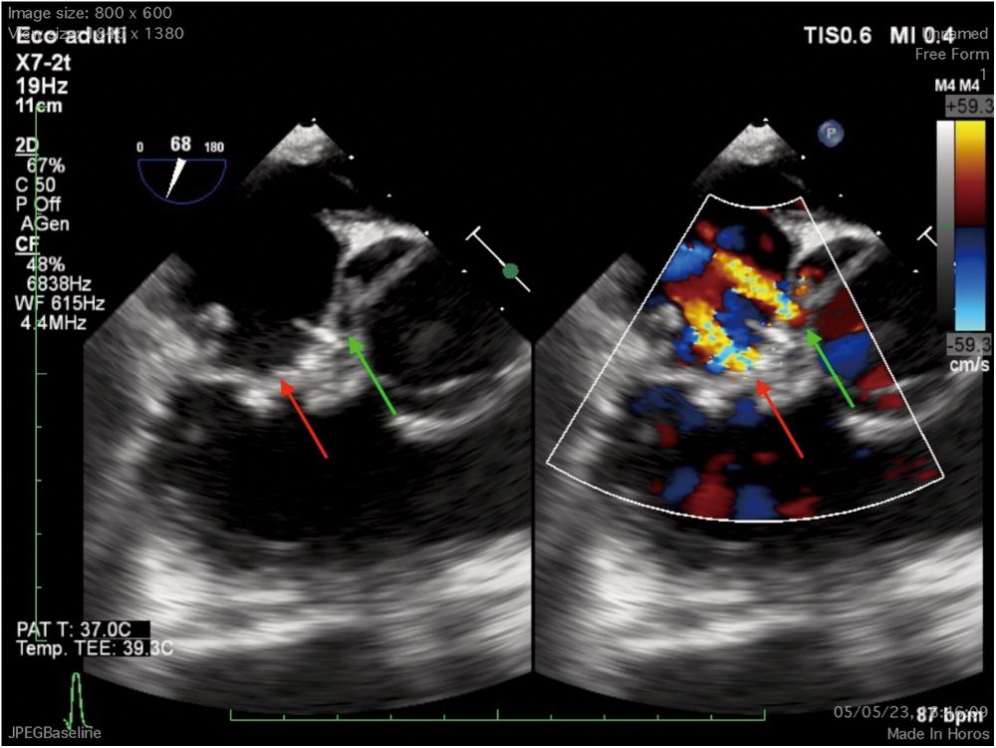
TEE (short axis) post-operative examination, 15 days after the surgery. Tricuspid regurgitation (red arrow) and aorta-right atrium’s shunt (green arrow) studied with colour Doppler.

**Figure 5:**
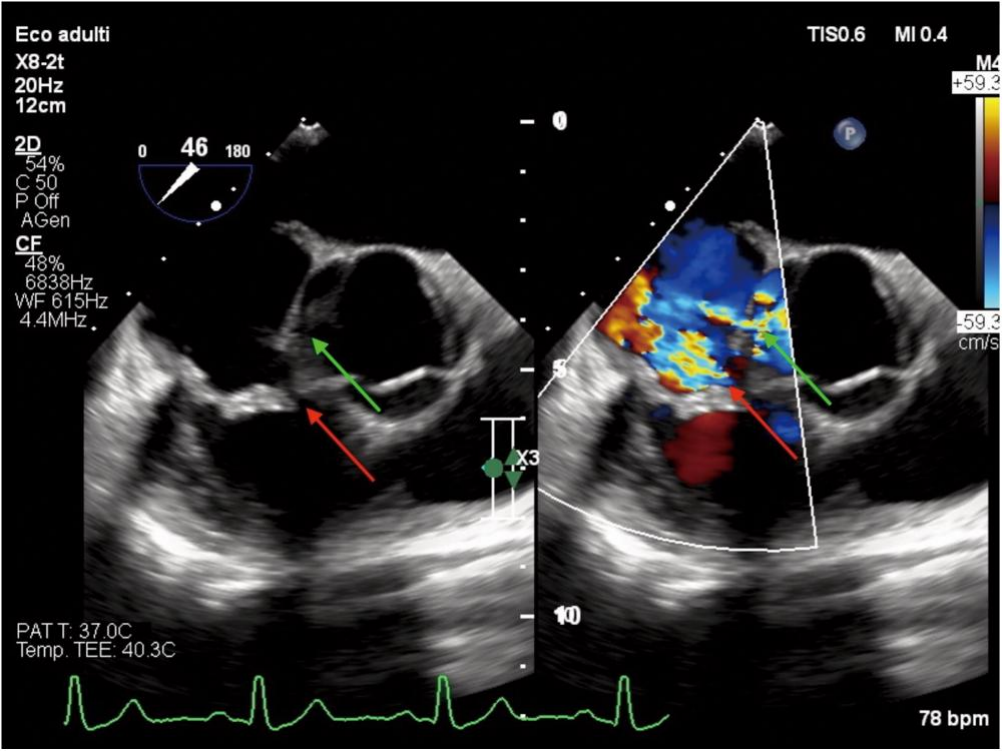
TEE (short axis), tricuspid regurgitation (red arrow) and aorta-right atrium’s shunt (green arrow) studied with colour Doppler. Two months after discharge, we can see result comparable to the post-operative examination.

A decision for TEE-guided cardiac surgery was made, including anterior flap reconstruction with autologous pericardium and insertion of Gore-Tex cord on its free edge. Finally, closure of the fistula between the left ventricle and right atrium was performed with two U-shaped prolene stitches with pledgets of untreated autologous pericardium. The mass had a cauliflower appearance; it appeared 3 × 4 cm in diameter. Since the excised mass was clearly an infectious vegetation, it was not sent to histopathology but to the microbiology laboratory in the hope of discovering the responsible infectious agent. However, no organisms were detected on the microbiology of the tissue sample. The intervention was successful with minimal tricuspid insufficiency and normo-positioned patch with no shunt. The post-operative course was without complications. The TEE follow-up, 15 days after surgery, showed mild tricuspid insufficiency and mild left–right shunt (*Figure 4*). During the hospital stay, various blood cultures and serological examinations were performed with negative results, so the patient continued empirical therapy with beta-lactam and glycopeptide. With the addition of the antifungal agent (fluconazole 50 mg/day for 4 weeks) to medical therapy, the fever resolved. The patient was discharged 35 days after surgery and complete clinical recovery. He was referred for cardiac rehab post-discharge. Two months after discharge, the patient underwent a new echocardiographic follow-up that showed a result comparable to the post-operative examination (*Figure 5*).

## Discussion

Fistula is a rare complication of infective endocarditis, occurring in only 1.6% of cases, and represents one of the most feared consequences of uncontrolled infection.^5^ Indeed, patients who develop a fistula frequently require surgery (87%), with high post-operative mortality (41%).^5^ A particular feature of our case is the sequence of several negative blood cultures, highlighting a very rare case of complicated blood culture-negative infective endocarditis (BCNIE) affecting the tricuspid valve. This condition limited therapeutic options to empirical therapy and early surgery.

In our case, transoesophageal echocardiography (TEE) allowed the diagnosis of right-sided infective endocarditis and revealed the perivalvular spread of the infection, complicated by abscess formation and subsequent fistula formation between two chambers. Initially, with transthoracic echocardiography (TTE), we noted a possible tricuspid vegetation through the apical four-chamber view and suspected the presence of a shunt in the apical five-chamber and parasternal short-axis views. Finally, our suspicions were confirmed with TEE, using a mid-oesophageal window in the four-chamber view (0°) and an upper oesophageal window in the short-axis view (60°–90°), with the aid of colour Doppler and 3D imaging.

This case highlights the crucial role of echocardiography, from early diagnosis (a major criterion according to the modified Duke criteria) to intraprocedural guidance and post-operative follow-up.

## Lead author biography


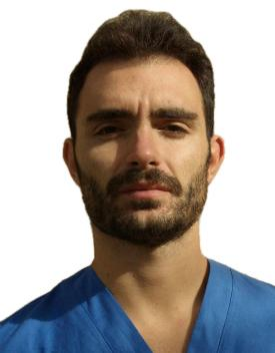


Giovanni Bellina is a medical doctor, currently a Cardiology resident at Catania’s University in Italy. He attended the University of Catania where he graduated with honours in 2020. He is passionate about clinical and cardiovascular imaging; he attends assiduously the cardiological intensive care unit and the cardiovascular imaging clinic of C.A.S.T.-Policlinico Hospital in Catania.

## Supplementary Material

ytae570_Supplementary_Data

## Data Availability

The data underlying this article are available in the article and in its online Supplementary material.

## Supplementary material

**Consent:** The authors confirm that the written consent for submission and publication of this case report, including images and associated text, has been obtained from the patient in line with COPE guidance.

**Funding**: None declared.
